# Type 1 IGF receptor associates with adverse outcome and cellular radioresistance in paediatric high-grade glioma

**DOI:** 10.1038/s41416-019-0677-1

**Published:** 2019-12-20

**Authors:** Aaron D. Simpson, Ying Wei Jenetta Soo, Guillaume Rieunier, Tamara Aleksic, Olaf Ansorge, Chris Jones, Valentine M. Macaulay

**Affiliations:** 1 Department of Oncology, University of Oxford, Old Road Campus Research Building, Roosevelt Drive, Oxford, OX3 7DQ UK; 2 Nuffield Department of Clinical Neurosciences, University of Oxford, Level 6, West Wing, John Radcliffe Hospital, Oxford, OX3 9DU UK; 3 Institute of Cancer Research, 15 Cotswold Road, Sutton, London, SM2 5NG UK; 4 Oxford Cancer and Haematology Centre, Oxford University Hospitals NHS Foundation Trust, Churchill Hospital, Oxford, OX3 7LE UK

**Keywords:** CNS cancer, CNS cancer

## Abstract

High-grade glioma (HGG) is highly resistant to therapy, prompting us to investigate the contribution of insulin-like growth factor receptor (IGF-1R), linked with radioresistance in other cancers. IGF-1R immunohistochemistry in 305 adult HGG (aHGG) and 103 paediatric/young adult HGG (pHGG) cases revealed significant association with adverse survival in pHGG, with median survival of 13.5 vs 29 months for pHGGs with moderate/strong vs negative/weak IGF-1R (*p* = 0.011). Secondly, we tested IGF-1R inhibitor BMS-754807 in HGG cells, finding minimal radiosensitisation of 2/3 aHGG cell lines (dose enhancement ratios DERs < 1.60 at 2–8 Gy), and greater radiosensitisation of 2/2 pHGG cell lines (DERs ≤ 4.16). BMS-754807 did not influence radiation-induced apoptosis but perturbed the DNA damage response with altered induction/resolution of γH2AX, 53BP1 and RAD51 foci. These data indicate that IGF-1R promotes radioresistance in pHGG, potentially contributing to the association of IGF-1R with adverse outcome and suggesting IGF-1R as a candidate treatment target in pHGG.

## Background

High-grade gliomas (HGGs) are treated with surgery, radiotherapy and in adults with concurrent and/or adjuvant temozolomide, but outcomes remain extremely poor.^1^ Adult HGG (aHGG) and paediatric HGG (pHGG) are distinct at the molecular level with histone *H3* gene mutation in ~50% of pHGG, while *IDH* and *EGFR* mutations are rare.^2,3^ Factors implicated in treatment resistance include amplified signalling via receptor tyrosine kinases (RTKs) including the type 1 insulin-like growth factor receptor (IGF-1R), which signals via phosphatidylinositol 3-kinase (PI3K)-AKT and extracellular signal-regulated kinases (ERKs) to promote proliferation and cell survival.^4^ IGF receptors and ligands are expressed in HGGs,^5^ and our data in other tumour types implicate IGF-1R in radioresistance via effects on the DNA damage response (DDR), influencing double strand break (DSB) repair by both non-homologous end-joining (NHEJ) and homologous recombination (HR).^6,7^ However, it is unclear whether IGFs contribute to radioresistance in HGG.

## Methods

### IGF-1R immunohistochemistry

HGG tissue microarrays (TMAs) were used for IGF-1R immunohistochemistry as described in Supplementary methods. Staining intensity was scored (0, negative; 1, weak; 2, moderate; 3, strong) by A.D.S. and T.A. and checked by O.A.

### Cell lines, reagents

We used aHGG cell lines GaMG, DK-MG, U87-MG and LN-18, and pHGG KNS42 and SF188. Cell line sources, mutation status, culture methods and reagents are described in Supplementary methods and Supplementary Table S1. Cells were treated with ionising radiation (IR) in a Gamma-Service Medical GmbH caesium-137 irradiator (GSR D1).

Western blotting, clonogenic assays, cell cycle analysis and immunofluorescence were performed as in ref.,^7^ using antibodies listed in Supplementary methods.

### Statistical analysis

Clinical survival data were censored at 99 months and Kaplan-Meier survival curves were analysed by Log-rank (Mantel-Cox) test. In vitro data analysis used Student’s *t*-test for two groups, with Holm-Šidák correction where multiple *t*-tests were applied, one-way analysis of variance (ANOVA) for >2 groups with one independent variable, two-way ANOVA for >2 groups with >1 variable, and Chi-square test for contingency tables. Dose enhancement ratios (DERs) were calculated as relative survival of controls/relative survival of IGF-1R-inhibited cells at specific IR doses. Data analysis used GraphPad Prism v7.0 or v8.0, all tests were 2-sided, and differences were considered significant if *p* < 0.05.

## Results

### IGF-1R expression associates with adverse outcome in paediatric HGG

**Fig. 1:**
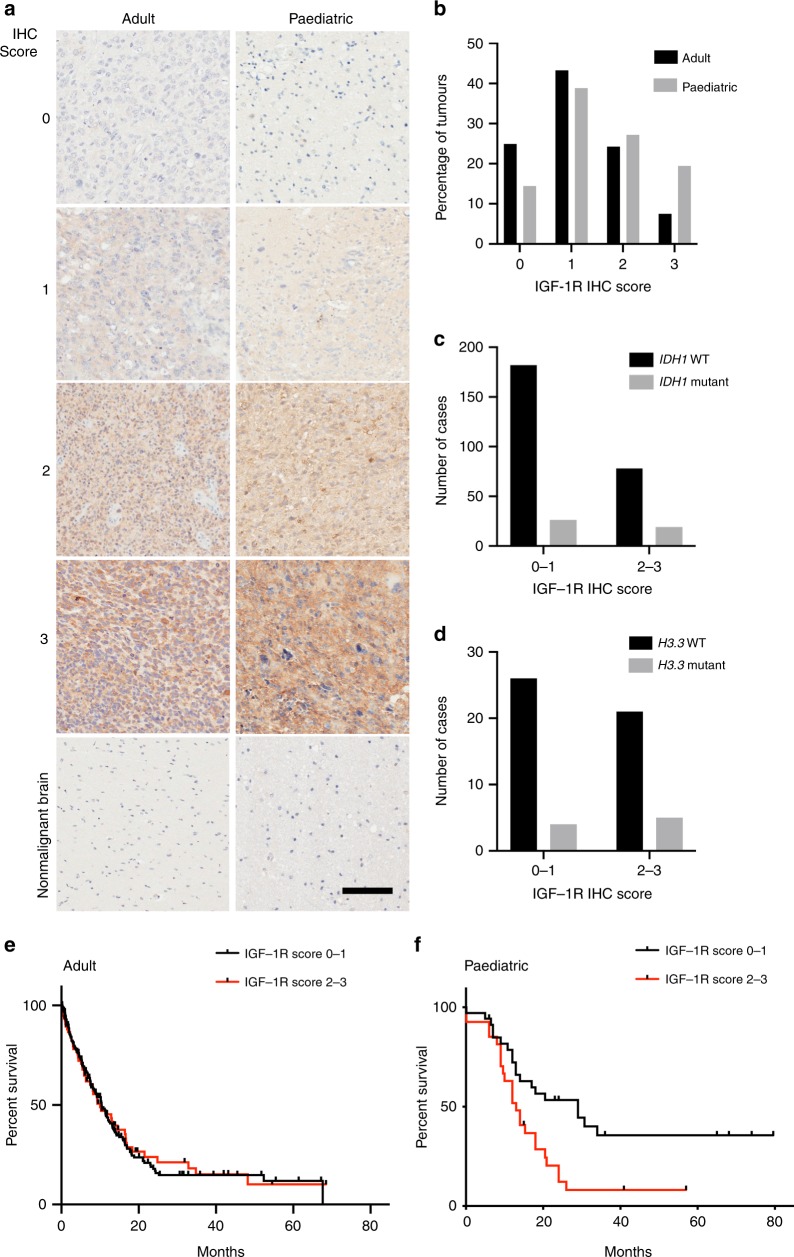
IGF-1R associates with adverse outcome in paediatric but not adult HGG. **a** Representative images of adult and paediatric HGGs scored 0–3 for IGF-1R intensity, with normal brain for comparison. Scale bar 100 μm. **b** Percentages of adult (*n* = 305) and paediatric (*n* = 103) HGGs scored 0–3 for IGF-1R staining intensity. **c** IGF-1R score by *IDH1*^*R132H*^ mutation status in aHGG. There were 45/305 cases with mutant *IDH1* including 26 of 208 cases (12.5%) with negative/weak IGF-1R (score 0-1) and 19 of 97 with moderate/strong IGF-1R (19.5%, *p* = 0.12, Chi-square test). **d** IGF-1R score by *H3.3* status in pHGG. The 47 wild-type *H3.3* pHGGs included 26 with negative/weak and 21 moderate/high IGF-1R, while the equivalent data for the nine *H3.3* mutant tumours were 4 and 5, respectively (*p* = 0.55, Chi-square test). **e**, **f** Kaplan-Meier survival curves for **e**, adult (*n* = 260) and **f**, paediatric patients (*n* = 65) by intensity of IGF-1R staining. There was no difference in outcome by IGF-1R score in the adult patients, with median overall survival of 10.4 months in those whose tumours scored negative/weak, and 10.0 months for those with moderate/strong IGF-1R tumours (*p* = 0.8251). Paediatric patients whose tumours scored 2–3 (moderate/strong) for IGF-1R had significantly shorter survival than those whose tumours scored 0–1 for IGF-1R (13.5 months vs 29 months, *p* = 0.011 by Log-Rank [Mantel-Cox] test).

We analysed 305 adult and 103 paediatric/young adult HGGs for IGF-1R, with eight adult and 11 paediatric non-malignant brain cores on the same TMAs. Most HGGs showed variable IGF-1R upregulation relative to normal brain, while IGF-1R was undetectable in 76 (24.9%) aHGGs and 15 pHGGs (14.4%; Fig. 1a, b). A higher percentage of aHGGs showed negative/weak IGF-1R, and pHGGs showed more frequent moderate/strong IGF-1R (Fig. 1b). The aHGGs had been analysed for *IDH1*^*R132H*^ mutation, and included 45/305 (15%) *IDH1* mutation positive and 260 (85%) mutation negative cases. There was a non-significant trend to increased *IDH1* mutation in moderate/strong IGF-1R tumours (*p* = 0.12, Fig. 1c). Clinical and/or molecular data (including *H3.3* but not *IDH* status) were available in 77/103 pHGGs, of whom nine harboured *H3.3* mutation (H3.3G34R in eight, H3.3K27M in one), 47 expressed wild-type *H3.3* (data unavailable in 21) with no difference in distribution of these cases between negative/weak and moderate/high IGF-1R tumours (*p* = 0.55, Fig. 1d), although numbers were very small. IGF-1R scores were analysed for associations with survival, for which data were available in 260 aHGG, of which 52 (20%) were stage III and 208 (80%) stage IV, and 65 pHGG including 21 (32%) stage III and 44 (68%) stage IV (Supplementary Table S2). Survival of aHGG patients did not vary with IGF-1R score (Fig. 1e). In the paediatric/young adult cohort there was evidence of improved survival for patients whose tumours had lower IGF-1R content (*p* = 0.011, Fig. 1f).

### IGF-1R inhibition suppresses HGG cell signalling and enhances pHGG radiosensitivity

**Fig. 2:**
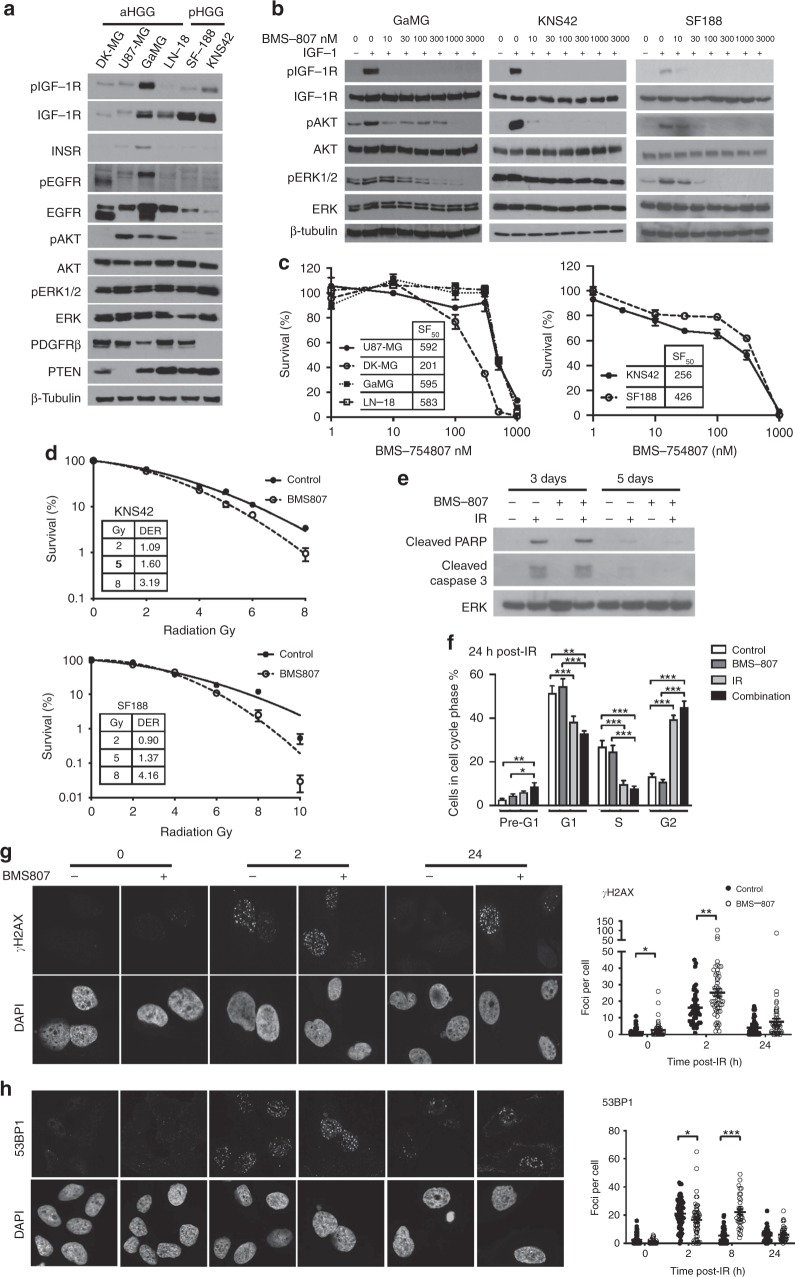
IGF-1R inhibition suppresses HGG cell survival and enhances radiosensitivity of pHGG cells. **a** Characterisation of RTK signalling in HGG cells. Western blots of whole cell extracts prepared from sub-confluent cultures in full medium with 10% FCS. Similar results were seen in 2–3 independently-prepared sets of cell extracts. **b** Serum-starved cells were treated with BMS-754807 or solvent control (0.015% DMSO) for 75 min and in the final 15 min with 50 nM IGF-1. Similar results were obtained in an independently-prepared set of cell extracts. **c** Effect of BMS-754807 on cell survival, expressed as mean ± SEM % survival of solvent-treated controls, pooled data from 2–3 independent experiments (6–9 data points) for aHGG and three independent experiments for pHGG cell lines. Legends show SF_50_ values, interpolated from the data as concentrations that suppressed cell survival to 50% of survival in solvent-treated controls. **d** Cells were pre-treated with solvent or 300 nM BMS-754807 (concentration selected from Fig. 2b-c as blocking IGF-1R while allowing sufficient survival to assess IR response) and 4 h later were irradiated. Graphs show pooled data from three independent assays in each cell line, mean ± SEM survival expressed as % survival in unirradiated dishes, with DER values. **e** SF188 cells cultured in full medium with 10% FCS were treated with solvent (control treatment) or 300 nM BMS-754807 for 4 h and irradiated (6 Gy). After 3 or 5 days, cells were lysed for western blot. Results are representative of three independent experiments. **f** SF188 cells were cultured, treated with solvent or BMS-754807 and irradiated as in **e**, and after 24 h collected for analysis by flow cytometry, showing mean ± SEM of five independent analyses (*p < 0.05, **p < 0.01, ***p < 0.001 by one-way ANOVA). **g**, **h**. SF188 cells were cultured, treated and irradiated as **e**, and fixed and stained at intervals for foci formed by: **g**, γH2AX; **h**, 53BP1. Left, representative images; right, graphs showing mean ± SEM foci per cell (*n* = 60–70 cells per condition from three independent experiments; *p < 0.05, **p < 0.01, ***p < 0.001).

Aiming to investigate the basis for association between IGF-1R and adverse outcome in pHGG but not aHGG, we utilised a panel of aHGG (DK-MG, U87-MG, GaMG, LN-18) and pHGG (SF-188, KNS42) cell lines. As in the clinical cancers, there was variable IGF-1R expression and we also noted variable AKT phosphorylation, while ERK phosphorylation was detected in all cell lines. We also detected variable expression of platelet-derive growth factor receptor-β and epidermal growth factor receptor (EGFR), including EGFR immunoreactivity at 140–150 kDa in DK-MG and GaMG that may represent EGFRvIII (Fig. 2a). To inhibit IGF-1R we used BMS-754807, which has reported IC_50_ values of 5–365 nM in human tumour cell lines.^8^ BMS-754807 inhibited IGF-1R phosphorylation in all cell lines, with AKT inhibition in all except U87-MG, consistent with its PTEN null status, and ERK inhibition in all but KNS42 (Fig. 2b, Supplementary Fig. S1A). BMS-754807 inhibited cell survival at ≥100 nM in aHGG cells and 10–30 nM in pHGG (Fig. 2c), but only DK-MG and KNS42 had SF_50_ values within the 5–365 nM range.^8^ To test effects on radiosensitivity, cells were pre-treated for 4 h with 300 nM BMS-754807 prior to irradiation. Supplementary Fig. S1B shows effects of 300 nM BMS-754807 (without irradiation) on cell survival. DK-MG cells formed ill-defined colonies, making radiation assays uninterpretable. Of the remaining cell lines, radiosensitivity was unaffected by BMS-754807 in U87-MG, while GaMG and LN-18 showed minor radiosensitisation, with DER values 1.3–1.6 at 2–8 Gy (Supplementary Fig. S1C). The pHGG cell lines KNS42 and SF188 were radiosensitised by BMS-754807 at IR doses >2 Gy, with DERs of 1.60 and 1.37 at 5 Gy and 3.19 and 4.16 at 8 Gy, respectively (Fig. 2d). We also found significant inhibition of cell survival and major radiosensitation of SF188 cells following siRNA-mediated IGF-1R knockdown (Supplementary Fig. S1D-F).

### IGF-1R inhibition influences the DDR in pHGG cells

Given the importance of IGF-1R for cell survival,^4^ we tested whether radiosensitisation was accompanied by apoptosis induction, but found no increase in cleavage of poly (ADP-ribose) polymerase (PARP) or caspase 3 post-irradiation of BMS-754807-pre-treated SF188 cells (Fig. 2e). Next, we performed cell cycle analysis, mindful that intrinsic radiosensitivity varies with cell cycle distribution, cells being most radiosensitive in G2-M, less sensitive in G1 and least in S phase.^9^ At 24 h post-irradiation, control-treated SF188 cells accumulated in G2, with fewer cells in G1 and S phase, and no significant change upon BMS-754897 pre-treatment either at 4 h pre-IR, or 24 h post-IR (Fig. 2f, Supplementary Fig. S2A, B). Finally, we examined induction and resolution of DNA repair foci, initially assessing γH2AX that marks IR-induced DSBs.^10^ BMS-754807-treated SF188 cells contained more γH2AX foci than controls at 2 hr post-IR (*p* < 0.01), with a non-significant excess at 24 h (*p* = 0.074, Fig. 2g), providing initial evidence that IGF-1R inhibition influenced the DDR. We also quantified foci formed by TP53 binding protein-1 (53BP1), which is recruited to DSBs undergoing NHEJ.^10^ Control-treated cells formed 53BP1 foci with a peak at 2 h, resolving over 8–24 h to baseline levels, while BMS-754807-pre-treated cells contained fewer 53BP1 foci 2 h post-IR (*p* < 0.05) and more at 8 h (*p* < 0.001; Fig. 2h). Lastly, we assessed foci formed by RAD51, the recombinase required for HR, finding no difference in focus numbers 2–8 h post-irradiation, but an excess of RAD51 foci at 24 h in BMS-754807-treated cells (*p* < 0.05, Supplementary Fig. 2C).

## Discussion

This study had two principal findings. Firstly, IGF-1R expression was significantly associated with adverse outcome in pHGG but not in aHGG. Broadly, there are two potential explanations for this difference. Firstly, IGF-1R may genuinely have no prognostic significance in aHGG, reflecting genetic differences between aHGG and pHGG.^2^ Secondly, IGF-1R could have adverse prognostic significance in aHGG that we failed to detect. Indeed, in a study of 218 aHGGs where a similar percentage (64%) were IGF-1R positive compared with 75% here, IGF-1R did associate with adverse outcome.^5^ The discrepancy between these data and ours could relate to differences in proportion of stage III and IV disease, and/or unbalanced frequency of *IDH* mutation, which was not stated in ref.,^5^ while we found a trend to increased *IDH1* mutation frequency in the IGF-1R moderate/strong vs negative/weak tumours (19.5% vs 12.5%, *p* = 0.12). Given that *IDH* mutation associates with favourable clinical prognosis,^2^ it is possible that the greater proportion of *IDH*-mutant cases in our aHGG cohort might have masked any adverse influence of IGF-1R. To increase confidence in the nature of the association of IGF-1R with outcome, it will be beneficial to examine larger HGG case series.

Secondly, we found that pHGG cells were radiosensitised by IGF-1R inhibition and depletion. We noted that radiosensitisation did not track precisely with relative sensitivity to BMS-754807 as a single agent. Considering SF_50_ values, the two cell lines most sensitive to BMS-754807 were DK-MG, the only cell line expressing both wild-type *TP53* and *PTEN*, and KNS42 that shows *PIK3CA* copy number gain, factors known to influence response to IGF axis blockade.^4,11^ We quantified radiosensitisation at 2, 5 and 8 Gy, because 2 Gy is the standard single fraction dose, and 5 and 8 Gy have been assessed as hypofractionated radiotherapy for HGG.^1,12^ With the exception of GaMG (DER 1.3 at 2 Gy), none of the cell lines were radiosensitised by BMS-754807 at 2 Gy (Fig. 2d, Supplementary Fig. S1C), suggesting that IGF axis inhibition may be ineffective with standard 2 Gy fractionated radiation. Interest in hypofractionation has arisen from recognition that rapidly-growing tumours such as HGG may progress towards the end of standard radiotherapy courses due to tumour repopulation.^12^ Supporting potential relevance in the context of hypofractionation, BMS-754807 induced more marked radiosensitisation of pHGG cells at 5 and 8 Gy (Fig. 2d), DERs exceeding the value of 1.2 said to be of potential clinical relevance.^13^ These effects could be mediated via inhibition of PI3K-AKT, reported to influence response to radiation-induced DSBs.^14^ Consistent with this, IGF-1R inhibition did not radiosensitise U87-MG that harbours *PTEN* mutation, which occurs more frequently in clinical aHGG than pHGG.^2^ While BMS-754807 suppressed ERK phosphorylation in most cell lines, this effect may not have contributed to radiosensitisation, given that BMS-754807 induced radiosensitivity but not ERK inhibition in KNS42 cells (Fig. 2b, d). Of possible relevance here, KNS42 harbours the *H3F3A* G34V mutation (Supplementary Table S1) shown to upregulate MYCN^3^ and we note that MYC is reported to regulate ERK activity.^15^

Investigating potential mechanisms of radiosensitisation, we found no evidence that BMS-754807 increased IR-induced apoptosis of pHGG cells. Neither was there evidence of redistribution to a more radiosensitive cell cycle phase during BMS-754807 pre-treatment or for 24 h post-IR, although it may be informative to assess cell cycle distribution beyond 24 h. We did, however, find evidence of DDR perturbation, with an excess of γH2AX foci in IGF-1R-inhibited cells 2 h post-IR. This early difference and the altered kinetics of 53BP1 focus formation implicate NHEJ, which accomplishes rapid repair of simple IR-induced breaks.^16^ We also detected a modest excess of RAD51 foci 24 h post-IR, suggesting an effect on HR. These data are consistent with our previous report of defective NHEJ and HR in IGF-1R-inhibited cells, indicated by a similar early defect in γH2AX focus resolution, epistasis with NHEJ kinase DNA-PK, and impairment of NHEJ and HR in repair reporter assays.^7^

In summary, we report that IGF-1R associates with adverse prognosis in pHGG patients and contributes to radioresistance of pHGG cells in vitro. These data suggest that there may be merit in testing whether IGF axis blockade is capable of sensitising pHGG to hypofractionated radiotherapy in vivo, using an IGF-1R inhibitor that has been shown to cross the blood–brain barrier.^17^ Positive findings in such studies would support assessment of this approach in early phase clinical trials for pHGG patients.

## Supplementary information

Supplementary file

## Data Availability

Data are available from the corresponding author on request.
