# Supplementary material for: Type 1 IGF receptor associates with adverse outcome and cellular radioresistance in paediatric high-grade glioma
Source: Br J Cancer. 2019 Dec 20;122(5):624–9. doi: 10.1038/s41416-019-0677-1 (PMC7054265; doi:10.1038/s41416-019-0677-1)
Supplement: Supplementary file 1 — Supplementary file [file 41416_2019_677_MOESM1_ESM.pdf]

Type 1 IGF receptor associates with adverse outcome and cellular radioresistance in paediatric high grade glioma.

Aaron D. Simpson, Ying Wei Jenetta Soo, Guillaume Rieunier, Tamara Aleksic, Olaf Ansorge, Chris Jones, Valentine M Macaulay

Supplementary Methods

IHC HGG tissue microarrays (TMAs) and clinical data were obtained with written consent as described [1, 2], and used with approval of National Research Ethics Service Committee South Central – Oxford C (reference 07/H0606/120). IGF-1R immunohistochemistry used IGF-1R antibody #9750 (Cell Signaling Technology) as described [3], and staining intensity was scored (0, negative; 1, weak; 2, moderate; 3, strong) by AS and TA and checked by OA. Tumours were scored for total IGF-1R intensity, not separately for membrane, cytoplasmic and nuclear IGF-1R as we had done previously [3], because plasma membranes and cytoplasm were indistinguishable, and nuclear IGF-1R was undetectable.

Cell lines: GaMG and DK-MG aHGG cell lines were from The Leibniz Institute DSMZ, U87-MG was from Adrian Harris (University of Oxford, UK) and LN-18 via Geoff Higgins from Ian Tomlinson (University of Oxford). KNS42 and SF-188 pHGG cell lines were obtained as [4, 5]. Cell lines were authenticated by STR genotyping (Eurofins Medigenomix Forensik GmbH). U87-MG, GaMG, and LN-18 were cultured in Dulbecco's Modified Eagle Medium (DMEM, Gibco), KNS42 and SF-188 in DMEM-F12 (Lonza) and DK-MG in RPMI-1640 (Gibco). Media were supplemented with 100 units/ml penicillin, 100 μg/ml streptomycin (Gibco) and 10% fetal calf serum (FCS, Gibco), and GaMG medium with 4X MEM non-essential amino acids (Gibco). Cultures were maintained at 37°C in 5% CO_2 except GaMG (10% CO_2). All cultures were negative when tested for mycoplasma (MycoAlert™ Kit, Lonza).

Reagents: We used long R3-IGF-1 (Sigma-Aldrich), IGF-1R/insulin receptor (INSR) inhibitor BMS-754807 (Selleck Chemicals) and IGF-1R siRNA (Hs_IGF1R_1) and non-silencing AllStars siRNA (Qiagen).

Western blotting used antibodies to IGF-1R (#3027), insulin receptor (#3025), phospho-Y1135/6-IGF-1R (#3024), EGFR (#2232), phospho-Y1068-EGFR (#2236), PDGFR (#3169), phospho-S473-AKT (#4058), AKT (#9272), phospho-T202/Y204 ERK1/2 (#9101), ERK1/2 (#4696), PTEN (#9559), cleaved PARP (#5625), caspase 3 (#9662, all from Cell Signaling Technology) and β-tubulin (#T4026, Sigma).

Clonogenic assays: Single cells were seeded at 3000 or 5000 cells/10cm dish or 1500 cells/well in six-well plates. The following day triplicate dishes/wells were treated with IR, BMS-754807 and/or solvent control (0.015% DMSO). Dishes/plates were incubated for 10-12 days (U87-MG, GaMG, LN-18), 8-10 days (SF-188), or 17-19 days (DK-MG, KNS42) until formation of >50 cell colonies, which were fixed, stained and counted on an automated counter (GelCount, Oxford Optronix).

Cell cycle analysis. Following 4hr pre-treatment with BMS-754807 (or solvent) and/or 6 Gy irradiation, cells were pulsed with 20 µM 5-bromo-2'-deoxyuridine (BrdU; B5002, Sigma) for 30 min. Adherent and floating cells were stained as described [6] with anti-BrdU antibody (B44, BD Biosciences) and Alexa Fluor 488 conjugated anti-mouse secondary antibody (Life Technologies). Cells were resuspended in PBS containing 10 µl/ml propidium iodide (Sigma-Aldrich) and analysed on a BD FACSCalibur (BD Biosciences), using FlowJo (v10) software.

Immunofluorescence. Cells were treated with 300 nM BMS-754807 or solvent, after 4hr irradiated (6 Gy) and stained for DNA damage foci as described [6], using antibodies to γH2AX (#05-636, Millipore), RAD51 (sc-8349, Santa Cruz) or 53BP1 (NB100-304, Novus Biologicals), with goat anti-mouse Alexa Fluor 488 secondary antibody. Images were acquired on a Zeiss 780 confocal microscope.

Supplementary references

1. Little SE, Popov S, Jury A, Bax DA, Doey L, Al-Sarraj S, et al. Receptor tyrosine kinase genes amplified in glioblastoma exhibit a mutual exclusivity in variable proportions reflective of individual tumor heterogeneity. Cancer Res. 2012;72(7):1614-20.
2. Mackay A, Burford A, Carvalho D, Izquierdo E, Fazal-Salom J, Taylor KR, et al. Integrated Molecular Meta-Analysis of 1,000 Pediatric High-Grade and Diffuse Intrinsic Pontine Glioma. Cancer Cell. 2017;32(4):520-37 e5.
3. Aleksic T, Gray NE, Wu X, Rieunier G, Osher E, Mills J, et al. Nuclear IGF-1R interacts with regulatory regions of chromatin to promote RNA polymerase II recruitment and gene expression associated with advanced tumor stage. Cancer Res. 2018:epub 7 May.
4. Bax DA, Little SE, Gaspar N, Perryman L, Marshall L, Viana-Pereira M, et al. Molecular and phenotypic characterisation of paediatric glioma cell lines as models for preclinical drug development. PLoS One. 2009;4(4):e5209.
5. Bielen A, Perryman L, Box GM, Valenti M, de Haven Brandon A, Martins V, et al. Enhanced efficacy of IGF1R inhibition in pediatric glioblastoma by combinatorial targeting of PDGFRalpha/beta. Mol Cancer Ther. 2011;10(8):1407-18.
6. Chitnis MM, Lodhia KA, Aleksic T, Gao S, Protheroe AS, Macaulay VM. IGF-1R inhibition enhances radiosensitivity and delays double-strand break repair by both non-homologous end-joining and homologous recombination. Oncogene. 2014;33(45):5262-73.
7. Memmel S, Sukhorukov VL, Horing M, Westerling K, Fiedler V, Katzer A, et al. Cell surface area and membrane folding in glioblastoma cell lines differing in PTEN and p53 status. PLoS One. 2014;9(1):e87052.
8. Rouillard AD, Gundersen GW, Fernandez NF, Wang Z, Monteiro CD, McDermott MG, et al. The harmonizome: a collection of processed datasets gathered to serve and mine knowledge about genes and proteins. Database (Oxford). 2016;2016.
9. Tate JG, Bamford S, Jubb HC, Sondka Z, Beare DM, Bindal N, et al. COSMIC: the Catalogue Of Somatic Mutations In Cancer. Nucleic Acids Res. 2019;47(D1):D941-D7.
10. Burford A, Little SE, Jury A, Popov S, Laxton R, Doey L, et al. Distinct phenotypic differences associated with differential amplification of receptor tyrosine kinase genes at 4q12 in glioblastoma. PLoS One. 2013;8(8):e71777.

	Cell line	Profile
aHGG	DK-MG	TP53 WT, PTEN WT, IDH1 WT
	GaMG	TP53 mutation, IDH1 WT, PTEN WT
	LN-18	TP53 mutation IDH1 WT, PTEN WT
	U87-MG	TP53 WT, IDH1 WT PTEN mutation
pHGG	KNS42	*TP53* mutation*, PIK3CA* copy number gain, *RB1* deletion, *H3F3A* G34V mutation.
	SF188	*TP53* mutation, *CCND1* and *CDK4* amplification, *NF1* deletion. *H3F3A* WT

Supplementary Table S1. HGG cell line mutation status.
Data from [4, 7] and the COSMIC (https://cancer.sanger.ac.uk/cosmic) and Cancer Cell Line Encyclopedia databases [8, 9].

	aHGG	pHGG
N	260	65
Age years: median (range)	58.4 (26.7 – 82.8)	13.2 (0.4 – 31.0)
Gender M/F/NA	140/101/NA	39/26
WHO* grade III/IV	52/208	21/44
H3.3 status WT/mutant/NA	N/A	39/8/18
IDH status WT/mutant 1/2	219/41/0	N/A
Survival months: median (range)	7.4 (0.1 – 67.6)	15.3 (0 – 197.9)

Supplementary Table S2. Demographic data for adult and paediatric/young adult HGG cases. Data are shown for 260 adult and 65 paediatric/young adult HGG cases in whom survival data were available. *Pre-2016 WHO classification criteria. NA, data not available. Patients in the paediatric / young adult cohort were aged <28 years, with one 31yr old included because the tumour harboured H3.3G34R [2]. *IDH1*^{R132H} mutation status had been ascertained in [10].

Supplementary Figure S1

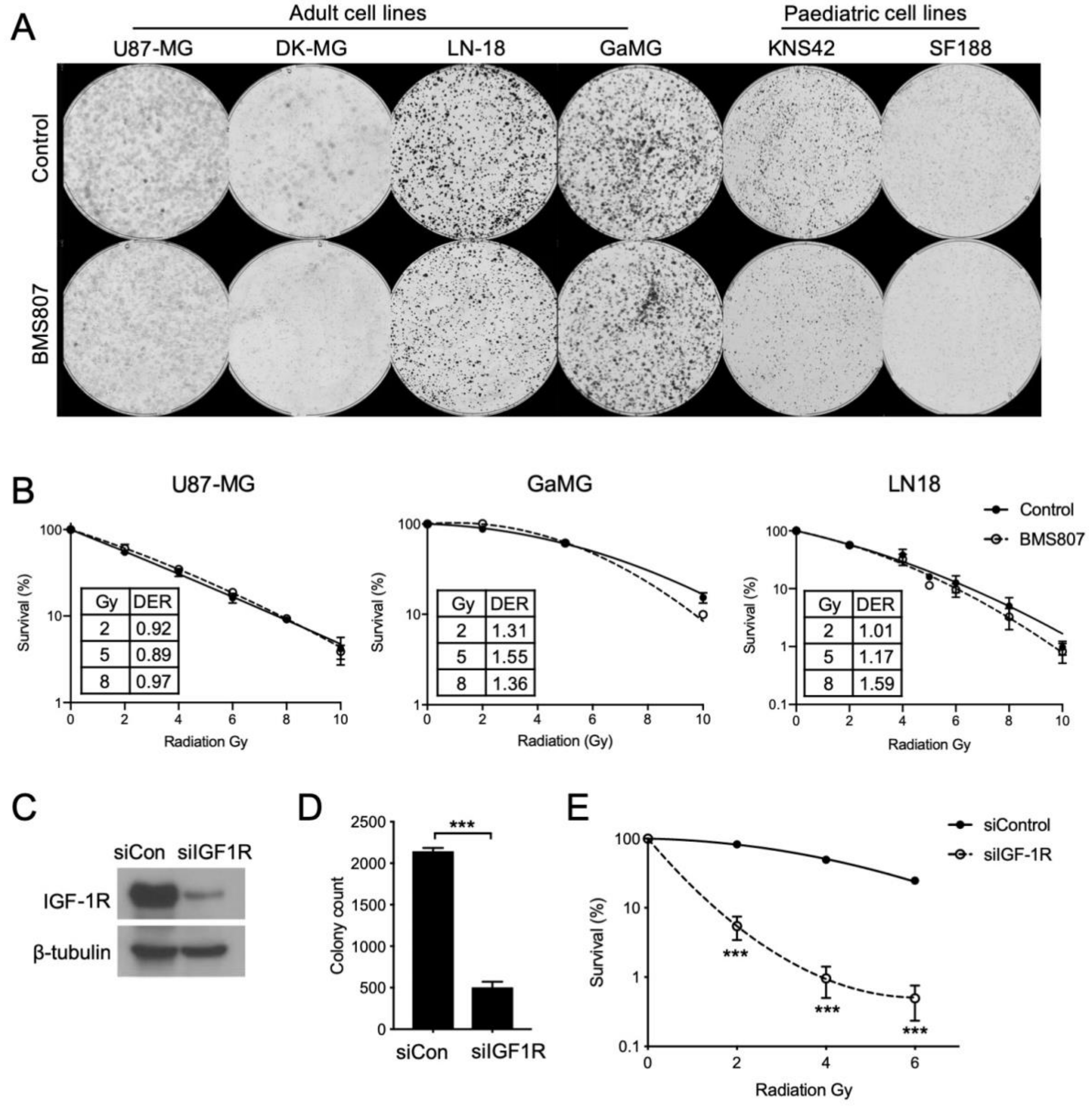

Supplementary Figure S1. IGF axis targeting enhances radiosensitivity of pHGG but not aHGG. A. Representative dishes from clonogenic assays testing effect of 300 nM BMS-754807 on HGG cell survival. **B.** Cells were pre-treated with solvent (0.015% DMSO) or 300 nM BMS-754807 and 4 hr later were irradiated. Graphs show pooled data from 3 independent assays in each cell line, mean ± SEM survival expressed as % survival in unirradiated dishes, with DER values. **C-E**. SF188 cells were transfected with 10 nM non-silencing control (siCon) or IGF-1R siRNA (siIGF1R) and after 48 hr were: C, lysed for western blot; D, re-seeded for clonogenic assay showing reduction in cell survival in IGF-1R depleted cells (***$p<0.001$); E, reseeded and the following day irradiated. Graph: pooled data, mean ± SEM % survival in unirradiated dishes, pooled data from two independent experiments (6 data points), showing marked enhancement of radiosensitivity at 2-6 Gy (***$p<0.001$). There were no surviving colonies in control or IGF-1R depleted dishes at IR doses >6 Gy.

Supplementary Figure S2

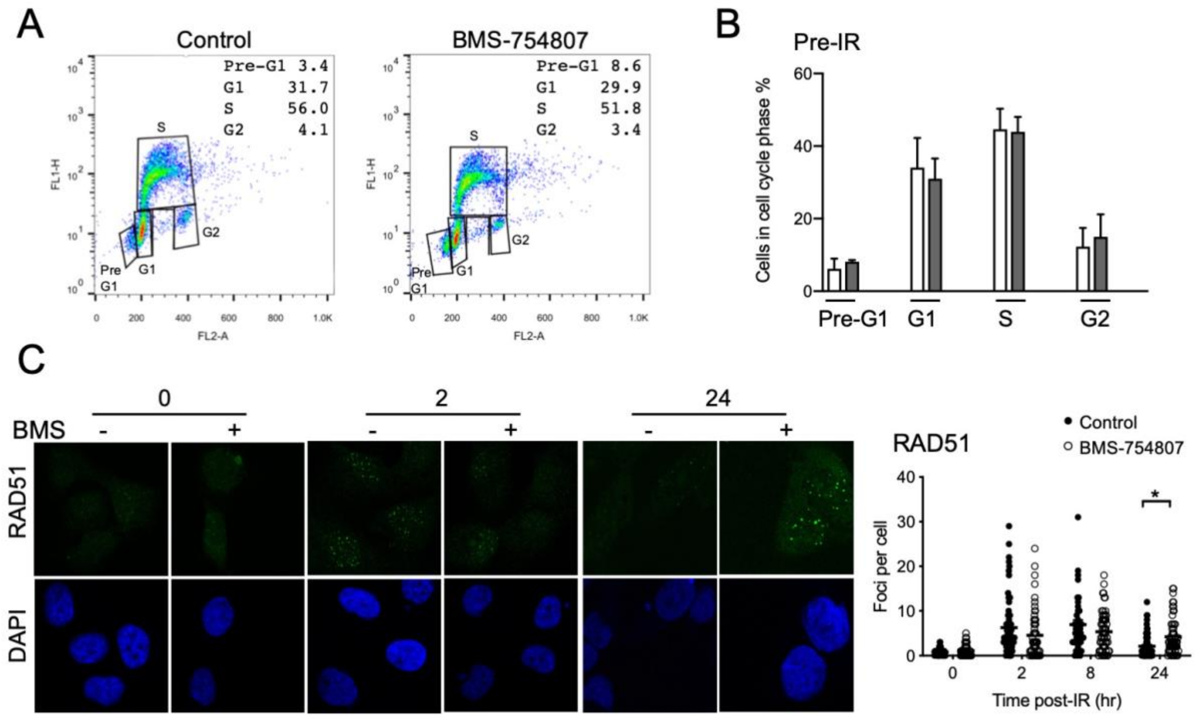

Supplementary Figure S2. Effects of IGF-1R inhibition on cell cycle distribution and RAD51 focus formation. A-B. SF188 cells were treated with solvent or 300 nM BMS-754807 and collected after 4 hr for analysis of cell cycle profile by flow cytometry. A shows representative scatter plot, B shows mean ± SEM of 3 independent analyses. **C.** SF188 cells were treated and irradiated as Figure 2G-H, and fixed and stained at intervals for RAD51 foci. Left, representative images; right, graphs showing mean ± SEM foci per cell (n=60-70 cells per condition from 3 independent experiments). At 24hr BMS-754807-treated cells contained more RAD51 foci than controls (4.2 ± 0.5 vs 2.1 ± 0.3, *$p<0.05$).
